# Outcomes and timing of laparoscopic cholecystectomy in gallstone disease: A single-center retrospective cohort study

**DOI:** 10.1097/MD.0000000000047900

**Published:** 2026-05-15

**Authors:** Turki Alhawiti, Akef Obeidat, Belal Nedal Sabbah, Mohammed Al-Rimawi, Mohammed Alfuwais, Abdulghaffar Khateeb, Nawaf Alharthi, Hussam Adi

**Affiliations:** a Thoracic Surgery & Lung Transplant, Lung Health Centre, Organ Transplant Center of Excellence (OTCoE), King Faisal Specialist Hospital & Research Center, Riyadh, Saudi Arabia; b College of Medicine, Alfaisal University, Riyadh, Saudi Arabia; c Laparoscopic and General Surgeon, Center King Salman Armed Forces Hospital at Northwestern Region, Tabuk, Saudi Arabia; d Center King Salman Armed Forces Hospital at Northwestern Region, Tabuk, Saudi Arabia.

**Keywords:** acute cholecystitis, gallstone diseases, laparoscopic cholecystectomy, postoperative complications, postoperative outcomes, retrospective cohort study, surgical timing

## Abstract

The optimal timing of laparoscopic cholecystectomy for gallstone disease remains debated. This study aimed to evaluate the outcomes of laparoscopic cholecystectomy and assess the impact of surgical timing on patient outcomes. We conducted a retrospective cohort study of 329 patients who underwent laparoscopic cholecystectomy at our institution. Demographic data, clinical presentations, surgical details, and outcomes were analyzed. The cohort was predominantly female (80.55%) with a mean age of 41.3 years. Most procedures (89.36%) were elective, with 43.47% of patients waiting over 4 months for surgery. Laparoscopic approach was successful in 98.78% of cases. Postoperative complications occurred in 5.47% of patients, with collection (2.43%) being the most common. Median surgery duration was 55 minutes. There was no significant association between waiting time and complication rates. Laparoscopic cholecystectomy demonstrates high success rates and low complication rates, even with extended waiting times. While timely intervention is ideal, our findings suggest that in resource-constrained settings, some delay in elective cholecystectomy may be acceptable for many patients without significantly increasing risks. Further prospective studies are needed to confirm these findings and explore patient-reported outcomes.

## 1. Introduction

Acute cholecystitis is a common inflammatory condition of the gallbladder, predominantly caused by gallstone-associated cystic duct obstruction.^[1]^ It affects a significant portion of the population, with typical presentations including right upper quadrant pain, nausea, and fever, often exacerbated by food intake.^[1]^ If left untreated, acute cholecystitis can lead to severe complications such as gangrenous or emphysematous cholecystitis, and in rare cases, gallbladder perforation.^[1]^

Laparoscopic cholecystectomy has become the gold standard treatment for acute cholecystitis, superseding open cholecystectomy due to its comparable efficacy, reduced morbidity, and lower costs.^[2]^ The timing of laparoscopic cholecystectomy, however, remains a subject of debate in the surgical community.^[2,3]^ Traditionally, patients have been categorized into 2 groups based on symptom duration: those presenting within 72 hours of symptom onset, who are candidates for early laparoscopic cholecystectomy (ELC), and those presenting after 72 hours, who are often managed conservatively with antibiotics before undergoing delayed laparoscopic cholecystectomy (DLC) after 4 to 6 weeks.^[3]^

Recent literature has questioned the practice of routinely delaying cholecystectomy beyond the 72-hour window.^[4]^ Several studies have suggested that ELC is associated with reduced postoperative complications, shorter hospital stays, and lower overall costs compared to DLC.^[5–7]^ However, these findings are not universally consistent across all studies, with some reporting contradictory results.^[8]^

The optimal timing for DLC, when ELC is not feasible, remains unclear. Some researchers propose that delaying surgery by at least 13 weeks may lead to fewer morbidities and postoperative complications, while others suggest that the time interval may be less critical than patient-specific risk factors.^[9]^ These conflicting findings highlight the need for further investigation into the optimal timing and outcomes of laparoscopic cholecystectomy. Furthermore, the realities of healthcare systems, including limited staffing and long surgical waiting lists, often necessitate delays in performing cholecystectomies.^[10]^ This practical constraint adds another layer of complexity to the clinical decision-making process and may influence patient outcomes.

Given these uncertainties and practical challenges, our study aimed to compare the outcomes of early versus delayed laparoscopic cholecystectomy in terms of postoperative complications, conversion rates, and length of hospital stay. Additionally, we sought to evaluate the impact of surgical waiting time on patient outcomes and identify patient-specific factors that may influence the choice between ELC and DLC.

## 2. Methods

### 2.1. Study design and setting

This retrospective cohort study was conducted at King Salman Armed Forces Hospital between January 1st, 2015 and December 31st, 2020. The study protocol was approved by the institutional ethics committee (HO-07-TU-002). This study is reported in line with the 2025 STROCCS guidelines.^[11]^

We included all patients aged 16 years and older who underwent laparoscopic cholecystectomy for gallstone disease during the study period. Patients were identified through the hospital’s electronic medical records system using procedure codes for laparoscopic cholecystectomy.

### 2.2. Data collection

Demographic data, past medical history, diagnostic information, and surgical details were extracted from patients’ medical records. Collected variables included demographic data (age, gender, and marital status), past medical history (presence of diabetes mellitus, hypertension, obesity, bronchial asthma, and other medical conditions), diagnostic data (time of diagnosis, stone number and size, history of cholangitis, acute cholecystitis, obstructive jaundice, biliary pancreatitis, and emergency room [ER] visits due to biliary colic), preoperative investigations (endoscopic retrograde cholangiopancreatography and magnetic resonance cholangiopancreatography [MRCP] results), and surgical data (nature of surgery, type of surgery, difficulty of surgery, waiting time for surgery, surgery duration, and postoperative complications).

### 2.3. Surgical procedure

All patients underwent laparoscopic cholecystectomy using a standardized four-port technique. The procedure was performed under general anesthesia by experienced surgeons or surgical trainees under direct supervision. Conversion to open cholecystectomy was done at the discretion of the operating surgeon when deemed necessary due to technical difficulties or complications.

### 2.4. Definitions

ELC was defined as surgery performed within 72 hours of symptom onset. DLC was defined as surgery performed after 72 hours of symptom onset. Difficult surgery was defined based on adhesions, anatomical variations, or prolonged operative time. Postoperative complications were categorized as bile leak, bile duct injury, retained stones, collection, and stent malposition.

### 2.5. Statistical analysis

Data were analyzed using Jamovi 2.6.26. Continuous variables were expressed as mean ± standard deviation or median (interquartile range) depending on the distribution of data. Categorical variables were presented as frequencies and percentages. Comparisons between groups (e.g., ELC vs DLC) were performed using Kruskal–Wallis test followed by Dunn test for continuous dependent variables which were segregated by categorical independent variables, as appropriate. A *P*-value <.05 was considered statistically significant.

### 2.6. Ethical considerations

Informed consent was waived due to the retrospective nature of the study. Patient confidentiality was maintained by using de-identified data for analysis.

## 3. Results

Our study included a total of 329 patients who underwent laparoscopic cholecystectomy. The demographic characteristics of the study population are presented in Table 1. The majority of patients were female (80.55%, n = 265), with males comprising 19.45% (n = 64) of the cohort. The age distribution showed that the largest proportion of patients (31.91%, n = 105) were in the 36 to 45-years of age group, followed by the 25 to 35 year age group (29.79%, n = 98). Marital status was known for 193 patients, of whom 91.19% (n = 176) were married.

**Table 1:** Demographic characteristics and past medical history of patients undergoing laparoscopic cholecystectomy (N = 329).

Demographic data	Total number (N = 329)		Percentage
**Gender**			
Male	64		19.45%
Female	265		80.55%
**Age**			
16–25	42		12.77%
25–35	98		29.79%
36–45	105		31.91%
46–55	53		16.11%
>55	55		16.72%
**Marital status**			
Single	17		5.17%
Married	176		53.50%
Unknown	136		41.34%
*Past medical data*	Total number (N = 329)	Percentage	
**Medical diseases**			
Medically free	281	85.41%	
DM	8	2.43%	
HTN	15	4.56%	
Obesity	2	0.61%	
Bronchial asthma	8	2.43%	
DM and HTN	2	0.61%	
DM, obesity, and bronchial asthma	1	0.30%	
DM, HTN, and Bronchial Asthma	1	0.30%	
Other medical conditions	10	3.04%	
**Previous surgeries**			
No previous surgeries	294	89.36%	
Underwent previous surgeries	35	10.64%	

Regarding past medical history (Table 1), the majority of patients (85.41%, n = 281) were medically free. Among those with preexisting conditions, hypertension was the most common (4.56%, n = 15), followed by diabetes mellitus and bronchial asthma (both 2.43%, n = 8 each). Most patients (89.36%, n = 294) had no previous surgeries.

The diagnosis of gallstone disease was made in various clinical settings (Table 2). The family medicine clinic was the most common site of initial diagnosis (41.64%, n = 137), followed by the ER (36.78%, n = 121). The majority of patients (75.99%, n = 250) had multiple gallstones, with stone sizes varying: 43.16% (n = 142) had stones <5 mm, while 41.03% (n = 135) had stones >10 mm.

**Table 2:** Diagnostic data and clinical presentations of patients undergoing laparoscopic cholecystectomy.

Diagnosis data	Total number (N = 329)	Percentage
**Time of diagnosis**		
Family medicine clinic	137	41.64%
General surgery clinic	53	16.11%
After attack of obstructive jaundice	18	5.47%
ER	121	36.78%
**Stone number**		
One	79	24.01%
Multiple	250	75.99%
**Stone size**		
<5 mm	142	43.16%
6–10 mm	52	15.81%
>10 mm	135	41.03%
**Cholangitis**		
Had an episode	12	3.65%
Did not have an episode	317	96.35%
**Acute cholecystitis**		
No attacks	289	87.84%
One attack	39	11.85%
Two attacks	1	0.30%
**Obstructive jaundice**		
No attacks	288	87.84%
One attack	35	10.64%
Two attacks	5	1.52%
Three or more attacks	1	0.30%
**Biliary pancreatitis**		
No episodes	296	89.97%
One episode	30	9.12%
Two episodes	3	9.1%
**ER visits due to biliary colic**		
No visits	225	68.39%
One visit	75	22.80%
Two visits	25	7.60%
Three or more visits	4	1.21%
**ERCP**		
Did not do	311	94.53%
Did once	17	5.17%
Did twice	1	0.30%
MRCP results		
Did not perform	267	81.16%
Normal result	40	12.16%
CBD stones	13	3.95%
Acute cholecystitis	8	2.43%
Acute pancreatitis	1	0.30%

CBD = common bile duct, ERCP = endoscopic retrograde cholangiopancreatography, MRCP = magnetic resonance cholangiopancreatography.

Regarding the clinical presentation and complications (Table 2), most patients did not experience severe complications prior to surgery. Only 3.65% (n = 12) had an episode of cholangitis, and 12.15% (n = 40) had at least 1 attack of acute cholecystitis. Obstructive jaundice occurred in 12.16% (n = 41) of patients, with the majority (10.64%, n = 35) experiencing a single episode. Biliary pancreatitis was observed in 10.03% (n = 33) of patients. Emergency room visits due to biliary colic were reported by 31.61% (n = 104) of patients, with most (22.80%, n = 75) visiting only once.

Preoperative investigations were selectively performed. Endoscopic retrograde cholangiopancreatography was conducted in 5.47% (n = 18) of patients, while MRCP was performed in 18.84% (n = 62) of cases. Among those who underwent MRCP, 12.16% (n = 40) had normal results, while 3.95% (n = 13) showed common bile duct (CBD) stones.

Regarding the surgical data (Table 3), the vast majority of procedures (89.36%, n = 294) were elective, with only 10.64% (n = 35) being emergent. Laparoscopic approach was successful in 98.78% (n = 325) of cases, with only 1.22% (n = 4) requiring conversion to open surgery. Most surgeries (93.01%, n = 306) were not classified as difficult.

**Table 3:** Surgical data and outcomes of laparoscopic cholecystectomy.

Surgery data	Total number (N = 329)	Percentage
**Nature of surgery**		
Emergent	35	10.64%
Elective	294	89.36%
**Type of surgery**		
Laparoscopic	325	98.78%
Open surgery	4	1.22%
**Difficult surgery**		
Difficult	23	6.99%
Not difficult	306	93.01%
**Time for surgery waited**		
<1 month	82	24.92%
1–2 months	36	10.94%
2–3 months	38	11.55%
3–4 months	30	9.12%
>4 months	143	43.47%
**Surgery duration**		
<1 hour	168	51.06%
1–2 h	141	42.86%
2–3 h	17	5.17%
>3 h	3	0.91%
**Post-op complications**		
No complications	311	94.53%
Bile leak	3	0.91%
Bile duct injury	1	0.30%
Retained stones	5	1.52%
Collection	8	2.43%
Stent malposition	1	0.30%

The waiting time for surgery varied considerably, with 43.47% (n = 143) of patients waiting >4 months for their procedure. The duration of surgery was <1 hour in 51.06% (n = 168) of cases, while 42.86% (n = 141) lasted between 1 to 2 hours.

Postoperative complications were relatively rare, occurring in 5.47% (n = 18) of patients. The most common complication was collection (2.43%, n = 8), followed by retained stones (1.52%, n = 5). Serious complications such as bile leak (0.91%, n = 3) and bile duct injury (0.30%, n = 1) were infrequent.

According to the registered data, there was a significant difference in general between time operated and postoperative complications. The statistically significant ones were all related to doing the surgery within a month of diagnosis. Bile leak, bile duct injury, and collections were all decreased in those patients with *P*-values of .298, .114, and .005 respectively. Interestingly, the time of surgery and the type of stone showed a statistically difference. Multiple stones and single stones are significant when the surgery was done in <1 month at a value of 0.019 and 0.007, respectively.

Upon analysis, there was no statistically significant difference between time of surgery and the following categories: Duration of surgery, type of surgery, MRCP results, and presence of cholangitis. Other comparisons were made between the different categories to determine statistical significance.

For example, a statistically significant difference was seen when surgery duration was compared to difficulty of surgery. Patients whose surgeries were <2 hours have been reported to have easy surgeries with a *P*-value of <.001. They have also been noted to have less complications similar to those mentioned earlier at *P*-values ranging from <.001 to .046.

Additionally, it is clear that there is a statistically significant difference between normal MRCP and lap cholecystectomy done in <1 hour at a level of 0.019 and evidence of CBD stone at a level of 0.002. It is also significant when the surgery is done between 1 to 2 hours and has evidence of CBD stone at a *P*-value of .048.

## 4. Discussion

This retrospective cohort of 329 patients provides valuable insights into the demographic characteristics, clinical presentations, surgical outcomes, and the impact of timing on laparoscopic cholecystectomy for gallstone disease.

The demographic profile of our cohort, with a predominance of female patients (80.55%) and a peak incidence in the 36 to 45 age group, aligns with established epidemiological patterns of gallstone disease.^[12]^ This gender disparity is often attributed to hormonal factors, particularly estrogen, which increases cholesterol saturation in bile.^[13]^

Our data reveal that the majority of patients (85.41%) were medically free, suggesting that gallstone disease often occurs in otherwise healthy individuals. However, the presence of comorbidities such as hypertension and diabetes mellitus in a subset of patients shows the need for comprehensive preoperative assessment and tailored perioperative management strategies.^[14]^

The high proportion of patients with multiple gallstones (75.99%) in our cohort is noteworthy. While the number of stones does not necessarily correlate with symptom severity, it may influence the technical difficulty of the procedure and the risk of complications such as CBD obstruction.^[14]^ Our analysis revealed a statistically significant difference in outcomes based on the timing of surgery, particularly for patients operated on within 1 month of diagnosis. These patients exhibited reduced rates of bile leak, bile duct injury, and collections (*P*-values of .298, .114, and .005, respectively). Additionally, the presence of multiple or single stones was significant when surgery was performed within 1 month (*P*-values of .019 and .007, respectively), suggesting that early intervention may mitigate complications associated with stone burden.

The relatively low incidence of severe complications such as cholangitis (3.65%) and biliary pancreatitis (10.03%) in our cohort may reflect effective triage and timely intervention. However, the fact that 31.61% of patients had at least 1 ER visit due to biliary colic indicates that many patients experience significant symptoms before definitive treatment. This finding raises questions about the potential benefits of earlier intervention in preventing recurrent episodes and emergency department utilization.^[4]^

Our surgical data reveal several important trends. The high success rate of laparoscopic cholecystectomy (98.78%) with low conversion rates to open surgery (1.22%) attests to the proficiency of our surgical team and the effectiveness of the laparoscopic approach in managing gallstone disease. This finding is particularly encouraging given that 10.64% of the procedures were performed emergently, situations that are often associated with higher conversion rates.^[15]^ Our analysis further showed that surgeries completed within 2 hours were significantly associated with fewer complications (*P*-values ranging from <.001–.046), reinforcing the importance of surgical efficiency in optimizing outcomes.

The distribution of waiting times for surgery in our cohort, with 43.47% of patients waiting >4 months, raises important questions about healthcare resource allocation and the potential impact of surgical delays on patient outcomes. While our study did not find a significant association between waiting time and overall complication rates, the analysis highlighted that early surgery (within 1 month) was associated with better outcomes for specific complications. This finding contributes to the ongoing debate about the optimal timing of cholecystectomy in non-emergent cases.^[16,17]^

Our findings align with some studies in the literature while contrasting with others. Lyu et al found that ELC reduces the rate of recurrent biliary pancreatitis and shortens hospital stays.^[18]^ Our analysis supports this, showing that early intervention may reduce certain complications. However, our low overall complication rates even with longer waiting times suggest that the benefits of ELC may not be universal, particularly in resource-constrained settings.

Interestingly, our results seem to contradict the findings of Rice et al, who reported higher hospital charges for same-admission cholecystectomy compared to delayed procedures.^[19]^ Our study did not analyze cost data, but the efficiency of our procedures (93.92% completed within 2 hours) suggests that cost differences might be institution-specific.

The low rate of difficult surgeries in our cohort (6.99%) is intriguing, especially considering the varied stone sizes and the presence of multiple stones in many patients. This contrasts with the findings of Lucocq et al, who reported increased perioperative morbidity with emergency cholecystectomy.^[20]^ Our results suggest that other factors, possibly including surgical expertise, may mitigate the expected difficulties in emergent cases.

Regarding the optimal timing for DLC, our findings seem to align more closely with Kourounis et al, who found no significant difference in outcomes between early and late interval laparoscopic cholecystectomy.^[21]^ This supports the notion that patient characteristics and risk factors may be more important than strict time intervals in determining surgical outcomes. However, our analysis underscores that certain complications, such as collections, are significantly reduced with earlier intervention, suggesting that timing may still play a critical role in specific contexts.

Our findings have several important implications for clinical practice and health policy. First, they reinforce the safety and efficacy of laparoscopic cholecystectomy across a diverse patient population. Second, they highlight the need for efficient referral pathways from primary care to reduce emergency presentations and potentially prevent recurrent symptoms. Third, they suggest that in resource-constrained settings, some degree of delay in elective cholecystectomy may be acceptable for many patients, provided that appropriate systems are in place for monitoring and emergency intervention if needed. However, the benefits of early intervention for reducing specific complications should not be overlooked.

Future research directions emerging from our study include the need for prospective investigations into the impact of surgical timing on patient-reported outcomes and quality of life, as well as cost-effectiveness analyses comparing different management strategies for gallstone disease. Additionally, exploring the factors contributing to the low rate of difficult surgeries in our cohort could yield valuable insights for preoperative risk stratification and surgical planning.

## 5. Limitations

This study has several limitations, as a retrospective, single-center study, it is susceptible to selection and information bias, and its generalizability may be limited. The lack of a control group or randomization precludes causal inferences about the relationship between surgical timing and outcomes. Long-term follow-up data and patient-reported outcomes were not collected, potentially underestimating complication rates and overlooking important measures of surgical success. The subjective categorization of surgical difficulty and the absence of data on confounding factors such as surgeon experience may have influenced our findings. Additionally, the small sample size in some subgroups limits our ability to draw robust conclusions about specific patient populations. Despite these limitations, our study provides valuable real-world data on laparoscopic cholecystectomy outcomes and contributes to the ongoing discussion about optimal management strategies for gallstone disease.

## 6. Conclusion

Our study suggests that laparoscopic cholecystectomy is a safe and effective procedure for the management of gallstone disease, with high success rates and low complication rates across a diverse patient population. The findings suggest that, in many cases, some degree of delay in elective cholecystectomy may be acceptable without significantly increasing the risk of adverse outcomes. However, the considerable proportion of patients presenting to emergency departments with biliary colic and the reduced complication rates associated with early intervention underscore the need for timely intervention and efficient referral pathways. The demographic and clinical characteristics observed in our cohort, including the predominance of middle-aged female patients and the high incidence of multiple gallstones, align with established epidemiological patterns and reinforce the importance of considering gallstone disease in the differential diagnosis of upper abdominal pain. While our study has limitations, including its retrospective nature and single-center design, it provides valuable real-world data that can inform clinical decision-making and resource allocation in the management of gallstone disease. Future prospective studies are warranted to further elucidate the optimal timing of cholecystectomy and to explore patient-reported outcomes in this common surgical procedure.

## Author contributions

**Conceptualization:** Turki Alhawiti, Akef Obeidat, Belal Nedal Sabbah, Mohammed Al-Rimawi, Mohammed Alfuwais, Abdulghaffar Khateeb, Nawaf Alharthi, Hussam Adi.

**Data curation:** Turki Alhawiti, Akef Obeidat, Belal Nedal Sabbah, Mohammed Al-Rimawi, Mohammed Alfuwais, Abdulghaffar Khateeb, Nawaf Alharthi, Hussam Adi.

**Supervision:** Turki Alhawiti, Belal Nedal Sabbah, Mohammed Alfuwais.

**Writing – original draft:** Turki Alhawiti, Akef Obeidat, Belal Nedal Sabbah, Mohammed Al-Rimawi, Abdulghaffar Khateeb, Nawaf Alharthi, Hussam Adi.

**Writing – review & editing:** Belal Nedal Sabbah.
